# Wnt Signaling in Leukemia and Its Bone Marrow Microenvironment

**DOI:** 10.3390/ijms21176247

**Published:** 2020-08-28

**Authors:** Yongsheng Ruan, Hye Na Kim, Heather Ogana, Yong-Mi Kim

**Affiliations:** 1 Department of Pediatrics, Division of Hematology, Oncology, Blood and Marrow Transplantation, Children’s Hospital Los Angeles, Norris Comprehensive Cancer Center, University of Southern California, Los Angeles, CA 90027, USA; yruan@chla.usc.edu (Y.R.); hyekim@chla.usc.edu (H.N.K.); hogana@chla.usc.edu (H.O.); 2 Department of Pediatrics, Nanfang Hospital, Southern Medical University, Guangzhou 510515, China

**Keywords:** Wnt signaling, leukemia, leukemia stem cell, leukemia microenvironment

## Abstract

Leukemia is an aggressive hematologic neoplastic disease. Therapy-resistant leukemic stem cells (LSCs) may contribute to the relapse of the disease. LSCs are thought to be protected in the leukemia microenvironment, mainly consisting of mesenchymal stem/stromal cells (MSC), endothelial cells, and osteoblasts. Canonical and noncanonical Wnt pathways play a critical role in the maintenance of normal hematopoietic stem cells (HSC) and LSCs. In this review, we summarize recent findings on the role of Wnt signaling in leukemia and its microenvironment and provide information on the currently available strategies for targeting Wnt signaling.

## 1. Introduction

Leukemia represents a heterogeneous group of blood neoplasia commonly characterized by an abnormal production of blood cells. Common leukemia subtypes include acute myeloid leukemia (AML), acute lymphoblastic leukemia (ALL), chronic myeloid leukemia (CML), and chronic lymphoblastic leukemia (CLL) [1,2,3,4]. Impressive therapeutic progress has been made within the last two decades, especially for ALL [3]; however, resistance to chemotherapy and a relapse of the disease remains a challenge.

The survival and maintenance of normal hematopoietic stem cells (HSC) relies on interactions with the bone marrow (BM) microenvironment, also known as BM niches [5]. Leukemic stem cells (LSC) may resist chemotherapy and give rise to a relapse of the disease, and may share many features with normal HSCs, including dependence on BM niches for their survival and resistance to chemotherapy [6,7]. The BM microenvironment consists of different components, including mesenchymal stem/stromal cells (MSCs) [8,9], endothelial cells [10], osteoblasts [11], peripheral neurons [12], and their associated nonmyelinated Schwann cells [13].

Studies suggest a fundamental role for the Wnt signaling pathway in HSC [14], cancer stem cells [15,16,17], and the tumor microenvironment [18,19]. In this review, we focused on the role of the Wnt signaling pathway in the microenvironment of leukemia stem cells (LSCs), major components of the leukemia microenvironment, and Wnt-signaling-targeting approaches.

## 2. Wnt Signaling Pathway

**Figure 1:**
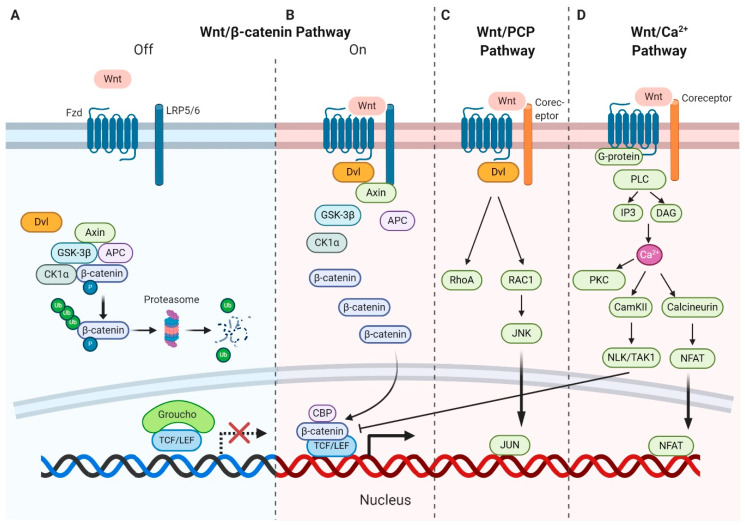
Wnt signaling pathways. Canonical Wnt–β-catenin pathway: (**A**) In the absence of Wnt (off state), the formation of the destruction complex, adenomatous polyposis coli (APC)–Axin–CK1α– glycogen synthase kinase 3β (GSK-3β) promotes β-catenin phosphorylation and degradation through the proteasome pathway. (**B**) In the presence of Wnt–Frizzled (Fzd) receptor interaction (on state) in association with the lipoprotein-receptor-related protein 5/6 (LRP5/6) coreceptor, the destruction complex is dissembled with promotion of β-catenin stabilization and nuclear translocation, triggering the expression of downstream Wnt target genes. Noncanonical Wnt pathways interact with Fzd and other coreceptors to recruit a disheveled segment polarity protein (Dvl). (**C**) In the Wnt–planar cell polarity (PCP) pathway, Dvl activates the small GTPases RhoA and RAC1, resulting in activating c-Jun *N*-terminal kinase (JNK). (**D**) Sidewise in the Wnt–Ca^2+^ pathway, activated phospholipase C (PLC) results in a cytosolic calcium flux, which, in turn, through several intermediate steps, promotes the nuclear factor of the activated T-cell (NFAT) transcription. ↓= Activation; ⊥ = Inhibition.

Wnt ligands are secreted glycoproteins that bind to Frizzled (Fzd) receptors, which are G-protein-coupled receptors (GPCRs) with seven transmembrane domains [20]. Upon binding, receptors activate multiple signaling pathways categorized as the canonical and noncanonical pathways [21,22]. The canonical pathway relies on the cytoplasmic stabilization of β-catenin (Ctnnb1), the key effector of Wnt signaling and a transcriptional coactivator for Wnt target genes [23]. In addition to Fzd receptors, Wnt ligands bind to the coreceptor lipoprotein-receptor-related protein 5/6 (LRP5/6) in canonical Wnt–β-catenin signaling [24]. In the absence of Wnt ligands, β-catenin is incorporated into the adenomatous polyposis coli (APC)–Axin–CK1α destruction complex, resulting in its phosphorylation by glycogen synthase kinase 3β (GSK-3β). These phosphorylation events mark β-catenin for subsequent degradation via the ubiquitin–proteasome pathway (Figure 1A).

Upon the binding of Wnt ligands to Fzd receptors and LRP5/6, a disheveled segment polarity protein (Dvl) is recruited to the intracellular domain of Fzd. Subsequently, Dvl acts as a platform for Axin to interact with the cytoplasmic domain of LRP5/6 and forms the foundation for the signalosome. This interaction disassembles the APC–Axin–CK1α–GSK3β degradation complex, leading to the accumulation and translocation of unphosphorylated β-catenin to the nucleus. In the nucleus, another multiprotein complex called the enhancesome forms when β-catenin displaces the corepressor Groucho and interacts with the T-cell factor/lymphoid enhancer factor (T-cell factor (TCF)/LEF) family of transcription factors to regulate the transcription of several target genes [25] (Figure 1B).

The dynamic polymerization of multiprotein assemblies such as the degradation complex in the presence of Wnt and the signalosome in the absence of Wnt is complex [26]. Dvl and Axin are key proteins for signaling in the assemblies and each contains a DIX domain. The DIX domain of Dvl polymerizes with Fzd and CK1, while the DIX domain of Axin polymerizes with domains from β-catenin, GSK-3β, and APC [27]. The two DIX domains of Dvl and Axin can also polymerize in the presence of Wnt. This dynamic polarization achieves high avidity by increasing the local concentration of signal effectors.

Wnt ligands also activate β-catenin-independent noncanonical pathways, including the planar cell polarity (PCP) pathway and Wnt–Ca^2+^ pathway [28]. In the Wnt–PCP pathway, Wnt ligands, including Wnt5a, Wnt7a, and Wnt11, bind to Fzd receptors and activate the small GTPases RhoA and Ras-related C3 botulinum toxin substrate 1 (RAC1) via the activation of Dvl. RhoA upregulates Rho kinase while activated RAC1 enhances c-Jun *N*-terminal kinase (JNK) expression, triggering the expression of downstream target genes (Figure 1C). The PCP pathway also regulates cytoskeletal rearrangements and actin polymerization, enabling cell migration [29]. During noncanonical Wnt–Ca^2+^ pathway [22], Wnt ligands bind to Fzd receptor and subsequent interact with coreceptor Ror1/2, initiating G-proteins, which in turn results in the activation of phospholipase C (PLC). Production of IP3 and DAG induced by PLC causes the release of calcium from the endoplasmic reticulum [30,31]. Calcium acts as a secondary messenger and further activates downstream proteins such as calmodulin-dependent kinase II (CamKII), calcineurin, and protein kinase C (PKC). CamKII activates NLK/TAK1 factors and NLK phosphorylates TCF/LEF, inhibiting the formation of the β-catenin–TCF–LEF complex. Calcineurin can trigger the transcription of target genes via the dephosphorylation of the nuclear factor of the activated T-cell (NFAT) and its transfer to the nucleus. PKC is involved in the PCP pathway via the activation of Cdc42 (Figure 1D). However, the role of noncanonical Wnt signaling in regulating the leukemia microenvironment remains largely unexplored.

Furthermore, Wnt signaling is regulated at different levels by a wide range of effectors including agonists and antagonists which act either intracellularly to modulate components of the signal transduction or extracellularly to modulate ligand–receptor interactions. Six families of secreted and four families of transmembrane Wnt antagonists are known to date: the Dickkopf proteins (Dkks) [32], secreted Frizzled-related proteins (sFRPs) [33], Wnt-inhibitory factor 1 (WIF-1) [34], Cerberus [35], Wise/SOST [36], insulin-like growth factor binding protein 4 (IGFBP-4) [37], Wnt-activated inhibitory factor 1 (Waif1/5T4) [38], adenomatosis polyposis coli downregulated 1 (APCDD1) [39], Tiki1 [40], and Shisa [41]. Two families of growth factors are known to activate Wnt signaling besides Wnts, Norrin [42], and R-spondins (RSPO) [43]. Of note, recently Wang et al. has reported that RSPO–leucine-rich-repeat-containing G-protein-coupled receptor 4 (LGR4) facilitated normal human hematopoiesis via TGF-β signaling activation [44]. In addition, a most recent study has demonstrated that targeting RSPO–LGR4 interaction using a clinical-grade anti-RSPO3 antibody (OMP-131R10/rosmantuzumab) impairs LSC self-renewal in AML patient-derived xenografts indicating a potential therapeutic role [45].

## 3. Wnt in HSC

Wnt signaling plays a critical role in the maintenance of HSC homeostasis [46,47]. To be precise, mild levels of Wnt activation enhance HSC function, whereas a high Wnt dosage impairs hematopoiesis, suggesting that canonical Wnt signaling regulates hematopoiesis in a dosage-dependent manner [48]. Wnt9a has served as a conserved regulator of zebrafish and human hematopoietic stem/progenitor cell (HSPC) development [49,50]. The Wnt9a-Frizzled-9b interaction in HSPCs is important for hematopoietic regulation and EGFR is required as a cofactor for the subsequent signal transduction [51]. Moreover, the noncanonical Wnt–Frizzled-6 signaling pathway has regulated HSPC expansion and survival in a hematopoietic-cell-intrinsic manner [52]. Histone deacetylase SIRT6 deletion has facilitated HSC proliferation through the aberrant activation of Wnt signaling through epigenetic modulation [53]. Another transcriptional repressor, Gfi1b, formed complexes with β-catenin and controlled both the cellularity and functional integrity of HSCs [54]. Hence, the HSC requires a precisely controlled level of activation of the Wnt signaling pathway for self-renewal, survival, development, and proliferation.

## 4. Wnt in CML LSC

It has been shown that the enhancement of self-renewal activity is associated with the activation of the Wnt–β-catenin pathway [55]. The *BCR–ABL1* oncogene, which plays a critical role in chronic myeloid leukemia (CML), can stimulate β–catenin in the blast phase of CML [56]. Moreover, the study, including both in vitro and in vivo methods, showed that the inhibition of the BCR-ABL–PI3K–AKT pathway promoted the degradation of β-catenin and reduced the transcription of β-catenin target genes, consequently reducing the tumor-initiating ability of CML cells in xenograft mice [56]. Meanwhile, nuclear β-catenin also promoted intrinsic tyrosine kinase inhibitor (TKI) resistance in CML progenitors [57]. Similarly, increased levels of nuclear β-catenin, Akirin-2, and NFκB-p65 proteins were found in imatinib-resistant CML cells [58]. In addition, the interaction of NFκB-p65 with FOXM1/β-catenin is important in CML leukemic stem cells (LSCs) [59]. It was revealed that the *FOXM1* gene is directly promoted by NF-κB and that the nuclear translocation of FOXM1/β-catenin requires NF-κB activation; meanwhile, Wnt–β-catenin activation facilitates the nuclear translocation of NFκB-p65 [59].

## 5. Wnt in AML LSC

Acute myeloid leukemia (AML) LSCs predominantly reside in the CD34^+^CD38^−^ fraction. Furthermore, CD34^+^ AML LSCs are primarily detected within lymphoid-primed multipotent progenitors (LMPP-like CD34^+^CD38^−^CD90^−^CD45RA^+^) and granulocyte-macrophage progenitor (GMP-like CD34^+^CD38^+^CD123^+^CD45RA^+^) subpopulations when xenografted into mice. Less frequently, they are detected in dominant multipotent progenitor (MPP-like CD34^+^CD38^−^CD90^−^CD45RA^−^) subpopulations. Interestingly, there are CD34^−^ AML LSCs that are primarily detected within a precursor granulocyte-macrophage (GM-like CD34^−^CD117^+^CD244^+/−^) subpopulation [60].

First of all, the Wnt–β-catenin signaling pathway was notably required for the self-renewal of LSCs in mouse models of AML [61]. Transcription factor FOXM1 and Six1/TCF7L2 upregulation activate Wnt–β-catenin signaling pathways by directly binding to β-catenin and stabilizing the β-catenin protein, thereby preserving LSC quiescence and promoting LSC self-renewal in MLL-rearranged AML [62,63]. Secondly, high levels of β-catenin were found in bone-marrow-resident leukemic cells in patients with FLT3-mutated AML [64]. Moreover, in another study, the inhibition of Wnt–β-catenin signaling synergized with FLT3 inhibitors in an FLT3-mutated AML mouse model [64]. Thirdly, β-catenin activation also has been shown to promote leukemia progression in vivo in xenograft mice reconstituted with del(5q) AML cell lines [65]. This effect can be abolished by the inhibition of β-catenin using indomethacin or shRNAs, resulting in the prolonged survival of del(5q) xenograft mice and a decrease of the proliferation of leukemia cells in the spleen and bone marrow (BM) [65].

Stem cell markers CD82 [66], CD70/CD27, and Wnt receptor Fzd1 are correlated in their proliferation and chemotherapy resistance for AML cells via activation of the Wnt–β-catenin signaling pathway. Overexpression of CD82 in the AML cell line THP1 accelerates the activation of the Wnt–β-catenin pathway [66]. A similar effect from CD70/CD27 was found in another study. The expression of CD70/CD27 in AML stem cells activated the canonical Wnt pathway and promoted AML cell proliferation [67]. Fzd1 knockdown has been shown to significantly reduce multidrug resistance 1 (MDR1) expression through inactivation of the Wnt–β-catenin pathway and to restore sensitivity to chemotherapeutic agents [68].

LEF1, as a central transcriptional mediator of Wnt signaling, has been able to cause AML in mice [69,70]. Additionally, inhibiting the binding of LEF1 to β-catenin impaired AML growth, in contrast to spared normal hematopoietic stem cells [71]. The serine-dephosphorylated form of Leo1, which is a direct and specific substrate of PRL-3, binds directly to β-catenin, promoting the nuclear accumulation of β-catenin and transactivation of TCF/LEF downstream target genes, such as *CCND1* (cyclin D1) and *C-MYC*, via noncanonical Wnt signaling [72].

As a synergistic strategy of targeting LSC and other applicable inhibitors, Wnt–β-catenin signaling plays an important role. For example, LSC-mediated resistance to BET inhibitors while Wnt–β-catenin signaling is inhibited, in turn, restores the sensitivity to BET inhibition [73].

## 6. Wnt in ALL

The Wnt–β-catenin pathway is upregulated in both B-cell-ALL (B-ALL) [74] and T-cell-ALL (T-ALL) [75,76]. It is noteworthy that β-catenin aberrantly activates β-catenin-dependent genes, including *AXIN1*, *C-MYC*, *BIRC5/Survivin*, *TCF1*, and *LEF1* in ALL [75,77,78,79]. An increase of β-catenin was found in LSCs of an in vivo mouse model of T-ALL after PTEN inactivation and c-Myc overexpression [80]. Similarly, recent studies have shown that c-Myc transcription is associated with leukemia-initiating cell capacity through the Wnt–β-catenin pathway in T-ALL [81,82]. Other than canonical Wnt–β-catenin signaling, Wnt5a ligand binding to ROR1 in TCF3-PBX1 B-cell precursor ALL cells activated noncanonical Wnt signaling [83]. It has also been described that Wnt5a–ROR1 interaction sustains TCF3-PBX1 cell proliferation through activation of the AKT–PI3K and STAT3 pathways [83].

## 7. Wnt in the BM Microenvironment 

### 7.1. Wnt in MSC 

There is accumulating evidence that functional alteration of stromal cells contributes to the development of myelodysplastic syndrome (MDS) and acute myeloid leukemia (AML) [84,85,86]. Haploinsufficient loss of β-catenin prevented the development of MDS in an *Apc^del/+^* mouse model in MSCs, showing that Wnt signaling in the BM niche is responsible for MDS, rather than hematopoietic cells [87]. Interestingly, an analysis of the gene expression profile of hMSC from AML patients (hMSC-AML) compared to healthy donors hMSCs (hMSC-HD) showed that BMP4 expression was decreased in hMSC-AML [88] and could be regulated by the Wnt signaling pathway [88] and could be regulated by the Wnt signaling pathway [89]. Fanconi anemia (FA)-AML-MSCs promoted the engraftment of healthy donor HSPCs and myeloid expansion of normal BM CD34^+^ cells in an in vivo mouse model via a COX2–PG-NR4A–Wnt signaling axis [90]. Moreover, this study showed that the upregulated NR4A–Wnt signaling axis was able to attenuate antileukemia immunity by inhibiting the production of leukemia-reactive CD8 cytotoxic T-lymphocytes [90].

MSCs also contribute to drug resistance through cell-adhesion-mediated drug resistance (CAM-DR) [91,92], in which the Wnt signaling pathway is involved [93]. The enhancement of N-cadherin in leukemia promotes adhesion to MSCs and leads to N-cadherin–β-catenin interaction [57,94]. In addition, the secretion of exogenous Wnt by MSCs activates the Wnt pathway in CML LSCs and mediates resistance to TKI treatment [95,96], whereas N-cadherin or H-cadherin blocking antibodies may abrogate the direct contact with BM stromal cells and the TKI resistance [57]. In CLL, Notch2 is activated in MSCs and subsequently induces a strong activation of canonical Wnt signaling in CLL cells [97]. Furthermore, the stabilization of β-catenin was mediated by not only the MSC-derived complement factor C1q, but also partially by stromal N-cadherin [97].

In vitro co-culture experiments showed that stromal cells provide acute leukemia cells with drug resistance through the upregulation of galectin-3 and stabilization of β-catenin [98]. Furthermore, this study highlighted that the upregulation of galectin-3 promoted Akt and GSK3β phosphorylation, revealing the relationship between galectin-3 and Wnt signaling [98]. Other studies have shown that MSCs protect ALL cells from drug-induced apoptosis via the downregulation of p21 expression and the activation of Wnt signaling pathways [99,100]. Likewise, CML MSCs protect tumor cells and increase their anti-apoptotic capability by activating the Wnt pathway [101]. In addition, leukemic bone marrow stromal cells exhibit aberrant Wnt3a and Wnt5a protein expression to promote the activation of both canonical and noncanonical Wnt signaling [102].

Studies have indicated that leukemic cells could alter the BM microenvironment and result in a bulk of growth factors to facilitate LSC propagation. First, it has been demonstrated that AML cells induced osteogenic differentiation in MSCs through activation of Smad1/5 signaling [103]. Moreover, a decreased expression of both SOX9 and EGR2 by RNA-Seq analysis was found in AML-MSCs compared to normal donor-MSCs inducing an increased adipogenic potential in AML-MSCs [104]. Similarly, another study by Shafat et al. showed that fatty acid-binding protein-4 (FABP4) messenger RNA was upregulated in adipocytes and AML when in co-culture with adipocytes [105]. Even though the role of marrow adipose tissue (MAT) in malignant hematopoiesis is controversial, MAT is very sensitive to changes in the leukemia patient’s metabolic status and the modified MAT may impact leukemia cell survival, proliferation, and anti-leukemic therapy [106]. In MDS-MSCs, Falconi et al. reported that GSK3β was expressed less at both the mRNA and protein levels while β-catenin protein and several WNT target genes were downregulated [107].

Taken together, Wnt signaling is one of many critical pathways by which MSCs facilitate leukemia cell survival, proliferation, and drug resistance. Meanwhile, leukemia cells may modify the MSC to support themselves.

### 7.2. Wnt in Endothelial Cells (ECs) 

Both paracrine (EC-dependent) and autocrine (EC-independent) vascular endothelial growth factor (VEGF)–VEGF receptor (VEGFR) signaling pathways have been demonstrated to be important in a human leukemia model [108]. A recent study indicated that the inhibition of Suz12, a core component of polycomb repressive complex 2 (PRC2), resulted in increased PI3K/mTOR, VEGF, and Wnt signaling [109]. Angiogenesis not only provides access to nutrients and oxygen, but also a source of factors that facilitate the survival, proliferation, and chemoresistance of leukemia [110]. In addition, studies have demonstrated that endothelial cells are able to transdifferentiate to mesenchymal cells, which is referred to as endothelial–mesenchymal transition (EndMT) [111]. TGF-β and the Snail transcription factor are two important stimulators of EndMT via the Notch and Wnt signaling pathways, utilizing Fzd2, Fzd9, and Wnt5b in the induction process [112]. However, fewer studies have focused on leukemia. Canonical Wnt signaling regulates the impact of classical Hodgkin lymphoma cells on EC migration, sprouting, and tube formation [113]. One of the possible mechanisms is that the Wnt–β-catenin signaling pathway promotes the proinflammatory factor TNF-α [114].

### 7.3. Wnt in Osteoblasts 

Osteoblasts, which are derived from MSCs, are bone-forming cells that line the endosteum and act together with osteoclasts, the bone-reabsorbing cells, to maintain bone homeostasis [115]. The maintenance of HSC production is correlated with the interactions between N-cadherin on osteoblasts and β-catenin on HSCs and bone morphogenetic protein (BMP) signaling [116]. It has been shown that the Wnt signaling inhibits the differentiation of MSCs into chondrocytes and adipocytes while promoting osteoblastic differentiation [117,118]. Moreover, during MSC osteogenesis, Wnt11, Fzd6, SFRP2, and SFRP3 are upregulated, while Wnt9A and Fzd7 are downregulated, suggesting that canonical Wnt signaling functions in sustaining an undifferentiated state, whereas noncanonical Wnt signaling facilitates osteogenic differentiation [119]. For instance, Wnt7b activates the Wnt–Ca^2+^ pathway, resulting in the nuclear import of Nfatc1 and activation of Sox11, which induces osteoblast differentiation in human BM MSCs [120].

Moreover, taurine, an osteocyte metabolite, was shown to have a protective effect in osteocytes against cell death due to reactive oxygen species in the IDG-SW3 cell line. In this study, taurine also remarkably inhibited Dkk1, an inhibitor of the Wnt–β-catenin signaling pathway, although only at higher doses [121]. Similarly, the activation of the Wnt–β-catenin signaling pathway mediated osteoblast differentiation via hesperidin, a compound that has many pharmacological activities, such as anti-inflammatory and antioxidation activities [122], and osteogenic capacity by CD39, which is produced from gingiva-derived MSCs.

A recent study using the human osteoblastic cell line SaOS-2 showed that mineralization and downregulated osteoblast marker genes, including alkaline phosphatase, osteocalcin, and osterix, and that genes associated with the proosteogenic Wnt signaling pathway were significantly inhibited by TKIs [123]. C-type lectin domain family 11 member A (Clec11a), as a growth factor for hematopoietic progenitor cells, activates the expression of osteoblast-related gene transcripts, including *Alp*, *Runx2*, *LEF1*, and *Axin*, and is associated with the development of several cancers, such as leukemia [124,125]. It is noteworthy that osteopontin (Opn), when secreted by bone marrow osteoblasts, may participate in the negative regulation of HSC proliferation while protecting leukemia cells from apoptosis by binding to the receptor αvβ3 [126]. For example, the blockade of αvβ3 from 3D polystyrene scaffolds coated with osteoblasts and AML cells resulted in more sensitivity to chemotherapy [127] through downregulation of the AKT–mTOR–β-catenin pathways [128]. through downregulation of the AKT–mTOR–β-catenin pathways [128].

Taken together, these studies emphasize that nonautonomous Wnt signaling in leukemia can be dependent on cues from the BM microenvironment.

## 8. Targeting Wnt Signaling

Preclinical studies have indicated that Wnt signaling pathways can be targeted, and recent reviews have broadly discussed the applicable compounds in various cancers [18,129,130,131]. Herein, we discuss compounds for targeting Wnt signaling in leukemia, including (1) targeting upstream effectors (2) promoting β-catenin degradation, and (3) inhibiting β-catenin–TCF interaction (Table 1).

### 8.1. Upstream Effector Targeting 

The available targets in this section include Wnt ligands and Fzd receptors. Porcupine (PORCN) is a membrane-bound-O-acetyltransferase enzyme that palmitoylates Wnts, which is important for its interactions with Fzd receptors [129]. PORCN inhibitors such as WNT974 (LGK974) [132], ETC-159 [145], IWP-2 [146], and IWP2G9 [63] have been well investigated in the Wnt signaling pathway. WNT974, in combination with the TKI nilotinib, significantly enhanced the inhibition of proliferation and colony-forming potential of CML LSCs and provided a survival benefit in vivo.

Dickkopf-1 (DKK1), a soluble inhibitor of Wnt–β-catenin signaling, regulates Wnt signaling by binding to the Wnt coreceptor LRP5/6. Since the levels of Wnt3a, Wnt5b, Wnt10a, Wnt14, Wnt16, Fzd3, Fzd6, and LRP5/6 were significantly higher in a vincristine-resistant BALL-1 ALL cell line (BALL-1/VCR), soluble DKK-1 selectively suppressed the Wnt signaling pathway and sensitized the response of BALL-1/VCR to chemotherapy [133].

Wnt inhibitors such as CGX1321, Foxy-5, and ipafricept (OMP-54F28), and Fzd inhibitors such as OSTA101 and vantictumab (OMP18RS) have been evaluated in clinical trials in cancers other than leukemia [130]. Dvl inhibitors like FJ9 and 3289-8625, used to disrupt Wnt signaling, have been investigated in other cancers with results of cancer regression [18]. In brief, these inhibitors are currently in phase I studies and further studies are required.

### 8.2. Promoting β-Catenin Degradation

Related components in the cytoplasmic portion of Wnt signaling are applicable targets, such as Axin, Dvl, CK1, and GSK. Tankyrases, the members of the poly (ADP-ribose) polymerase (PARP) family of enzymes, mediate the ubiquitin-based proteasomal degradation of Axin [18]. Tankyrase inhibitor IWR-1, in combination with treatment with salinomycin (SAL), synergistically triggered SAL-induced differentiation of acute promyelocytic leukemia (APL) cells by inhibiting Wnt–β-catenin signaling [134]. Blocking the Wnt pathway via the β-catenin inhibitor XAV939 sensitized B-ALL cells to Ara-C chemotherapy and improved overall survival in a mouse model [100]. Both IWP2G9 and the tankyrase inhibitor IWR107 attenuated the development of MLL-AF9 fusion AML via disruption of Wnt–β-catenin–SIX1 signaling [63]. It is noteworthy that both XAV939 and the PORCN inhibitor LGK974 were able to abolish the morphine-induced aberrant Wnt–β-catenin protective effect in blast-phase CML cells [147].

The inhibition of CK1 using PF-670462 significantly prolonged the overall survival of a CLL mouse model and had synergistic effects with the B-cell receptor (BCR) inhibitor ibrutinib in both in vitro and in vivo CLL [135].

### 8.3. Inhibiting β-Catenin–TCF Interaction 

CREB-binding protein (CBP)/catenin antagonists were recently reviewed as a critical strategy to target LSCs [93]. XX-650-23, a CBP inhibitor, showed promising preclinical efficacy for increasing sensitivity to TKI dasatinib in ALL [140]. ICG-001 is a representative small-molecule inhibitor of Wnt–catenin signaling which specifically binds to the N-terminus of CBP instead of p300, thereby disrupting the interaction between CBP and β/γ-catenin [93]. ICG-001 was able to eradicate drug-resistant primary B-ALL and CML in combination with conventional therapy in vitro, and significantly prolonged the survival of a B-ALL mouse model [137]. ICG-001 also led to the loss of self-renewal capacity in leukemia-initiating cells of B-ALL [137] and CML and the downregulation of survivin [137,148]. The expression of survivin, which is a Wnt–CBP–β-catenin-regulated gene [149], is crucial during hematopoiesis [150], as well as leukemogenesis [137,151] and CML and the downregulation of survivin [137,148]. Targeting survivin using EZN-3042 has markedly improved chemotherapeutic response in ALL models [152,153]. Moreover, EZN-3042 was administered and halted in a phase I clinical trial, since the combination of EZN-3042 with intensive reinduction chemotherapy was not tolerated at a dose that led to the consistent downregulation of survivin expression [154]. Furthermore, ICG-001, in combination with ZSTK-474, a PI3K inhibitor, induced apoptosis in T-ALL [136]. Similarly, combination therapy of ICG-001 and VS-5584, which is a dual PI3K/mTOR inhibitor, significantly reduced the leukemic burden and prolonged the survival of mice transplanted with human PRL-3 high AML cells [138]. Inhibition by the CBP/β-catenin antagonist C-82/PRI-724 exerted potent activities against AML LICs and synergized with FLT3 inhibition in FLT3-mutant AML [64].

In addition, a synergistic increase of apoptotic effects and restored chemosensitivity from prednisolone treatment in B-/T-ALL cells were confirmed by using traditional chemotherapeutic drugs and iCRT14, a β-catenin/TCF inhibitor [142]. A LEF1/β-catenin inhibitor, CGP049090, and PFK115-584 decreased the expression of CTNNB1/LEF1 target genes c-myc, cyclin D1, and survivin in AML cell lines Kasumi-1 and HL-60 [141]. PKF115-584 also prevented and partially reversed leukemogenesis in T-ALL [81].

Furthermore, BHX (*N*-(4-hydroxybenzyl)-1,3,4-triphenyl-4,5-dihydro-1*H*-pyrazole-5-carboxa-mide), a novel canonical Wnt–β–catenin-signaling inhibitor, was recently evaluated in the CML cell line K562, showing inhibition of cell proliferation in a dose-dependent manner and the induction of apoptosis [143].

### 8.4. Nonspecific Modulation of Wnt Signaling

An increasing number of studies have demonstrated that compounds may exert their effects by modulating Wnt signaling. For example, ARV-825, a BET/BRD4 inhibitor targeting c-Myc, can downregulate Wnt–β-catenin signaling, thereby decreasing the CD34^+^CD38^−^CD90^−^CD45RA^+^ leukemic stem cell population in AML [155]. Pyrvinium pamoate, an anthelmintic drug approved by the Food and Drug Administration (FDA), has potent inhibitory effects on growth and survival in CML cells via inhibition of Wnt–β-catenin signaling [156]. Emodin and 3′-azido-3′-deoxythymidine (AZT) synergistically decrease proliferation and induce apoptosis in CML through the regulation of the Wnt–β-catenin–EGR1 pathways [157]. The methylation inhibitor decitabine and pan-histone deacetylase inhibitor panobinostat, in combination with chemotherapy, are also associated with the depression of the Wnt–β-catenin pathway in AML studies. SKLB-677, a new FLT3 inhibitor, has exhibited the ability to inhibit Wnt–β-catenin signaling in AML clinical trials [158].

Interestingly, cotreatment with celecoxib, a specific COX-2 inhibitor, and doxorubicin significantly inhibited cell proliferation and induced apoptosis in AML [159,160]. Sulindac is a nonsteroidal anti-inflammatory drug (NSAID) that is used for releasing arthritis-related pain. Besides inhibition of COX, sulindac showed anticancer properties through inhibition of Wnt/β-catenin signaling pathway in colon cancer [161,162], adenoma [163] and breast cancers [164,165]. Treatment of AML cell lines THP-1 and HL60 with sulindac sulfide and diclofenac induced apoptosis and differentiation, suggesting antileukemic properties of sulindac [166], yet its connection with Wnt inhibition has not yet been studied.

Niclosamide is an antihelmenthic drug that is known for modulation of Wnt signaling, along with other signaling pathways such as mTORC1, STAT3, NF-kB, and Notch pathways, in many types of cancers [167,168,169]. Niclosamide was shown to decrease survival and self-renewal capacity of CD34^+^ CML LSCs, while ex vivo exposure of CML CD34^+^ cells decreased long-term engraftment in mice. Furthermore, in vivo treatment of p-Niclosamide alone prolonged survival of CML mice compared to placebo group, and combination therapy of p-Niclosamide with imatinib further prolonged survival of CML bearing mice. Anti-leukemic potential of Niclosamide was achieved by interfering the interplay between p65 of NF-κB and FOXM1/β-catenin [59].

Lastly, clofazimine, an antileprosy drug, is known for its anti-cancer potential via inhibition of canonical Wnt signaling [170]. Clofazamine showed anti-leukemic potential in CML by modulating transcriptional activity of PPARγ and its subsequent interaction with p65 NF-κB to induce degradation. Degradation of p65 decreased transcription of myeloblastoma oncoprotein (MYB), which led to the downregulation of peroxiredoxin 1 (PRDX1) with enhanced reactive oxygen species (ROS) induced apoptosis [171].

## 9. Conclusions

**Figure 2:**
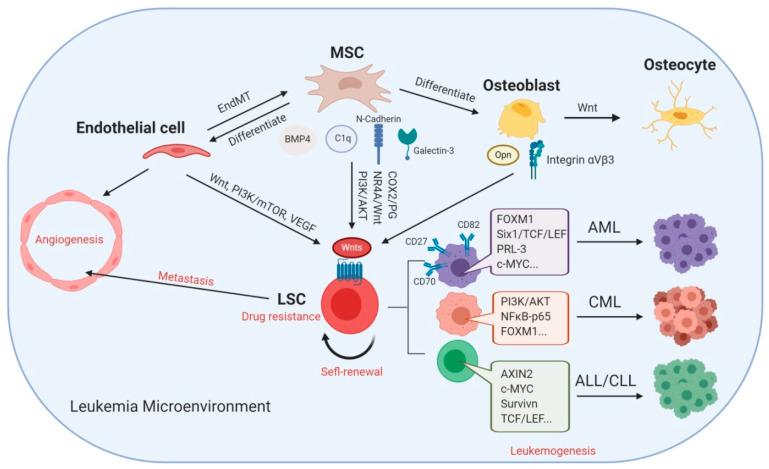
Aberrant Wnt signaling favors disease progression in leukemia. Mesenchymal stem/stromal cells (MSCs) not only directly produce Wnt, C1q, N-cadherin, and galectin-3 while decrease BMP4 initiating Wnt signaling in leukemia stem cells (LSCs), but also differentiate into endothelial cells and osteoblasts, which support LSCs as well. Activated Wnt signaling mediates LSC self-renewal and drug resistance, thereby promoting leukemogenesis. Endothelial cells are not only able to transdifferentiate to MSCs called endothelial–mesenchymal transition (EndMT) effect, but also form new blood vessels called angiogenesis by which LSCs metastasize. Wnt signaling facilitates osteoblast differentiation into osteocytes; meanwhile, osteoblasts promote drug resistance of LSC through osteopontin (Opn) and the integrin pathway. AML, acute myeloid leukemia; CML, chronic myeloid leukemia; ALL, acute lymphoblastic leukemia; CLL, chronic lymphoblastic leukemia.

Herein, we reviewed the complex relationship between Wnt signaling and many factors in the leukemia microenvironment (Figure 2). As discussed, most studies to date have been based on the canonical Wnt–β-catenin signaling pathway. Importantly, Wnt signaling pathways play a crucial role in sustaining the self-renewal potential of LSCs and regulating the components in the leukemia microenvironment, including MSCs, endothelial cells, and osteoblasts. It is noteworthy that most Wnt ligands are produced from MSCs, which result in the activation of Wnt signaling in LSCs. Interestingly, the interactions between MSCs and LSCs rely on cell-adhesion molecules, such as cadherins and integrins, and are involved in the process of Wnt signaling activation; therefore, CAM-DR plays a critical role in Wnt signaling. We summarized three groups of inhibitors targeting Wnt signaling pathways, which were (1) targeting upstream effectors, (2) promoters of β-catenin degradation, and (3) inhibitors of β-catenin–TCF interaction. However, Wnt signaling also plays a crucial homeostatic role in normal cells and restricts the administration of potent Wnt signaling inhibitors due to off-target toxicities. In the future, the dual targeting of Wnt signaling and the leukemia microenvironment may efficiently eradicate leukemia cells. Taken together, Wnt signaling exerts a critical effect in both leukemia stem cells and the leukemia microenvironment, which suggests that targeting the Wnt signaling pathways is a promising therapeutic strategy for leukemia.

## Figures and Tables

**Table 1:** Preclinical/Clinical Wnt–β-catenin Signaling Inhibitors in Leukemia.

Targets	Compound	Leukemia Type	Clinical Trial (Number)
(1) Targeting Upstream Effectors			
Porcupine (PORCN) inhibitors	WNT974 (LGK974)	Chronic myeloid leukemia (CML) [132]	Preclinical
PORCN inhibitors	IWP2G9	Acute myeloid leukemia (AML) [63]	Preclinical
DKK1	DKK1-conditioned medium	B-cell acute lymphoblastic leukemia (B-ALL) [133]	Preclinical
(2) Promoting β-catenin Degradation			
Tankyrase inhibitor	XAV939	B-ALL [100]	Preclinical
Tankyrase inhibitor	IWR-1	Acute promyelocytic leukemia (APL) [134]	Preclinical
Tankyrase inhibitor	IWR107	AML [63]	Preclinical
CK1 inhibitor	PF-670462	Chronic lymphoblastic leukemia (CLL) [135]	Preclinical
β-catenin degradation inhibitor	CWP232291	-	NCT01398462
(3) Inhibiting β-catenin–T-cell factor (TCF) Interaction			
CREB-binding protein (CBP)/catenin inhibitor	ICG-001	T cell (T)-ALL [136] B-ALL [137] AML [138] CML [139]	Preclinical
CBP/β-catenin inhibitor	PRI-724(C-82 pro-drug)	AML [64]	NCT01606579 NCT02195440
CBP inhibitor	XX-650–23	ALL [140]	Preclinical
LEF1/β-catenin inhibitor	CGP049090	AML [141]	Preclinical
LEF1/β-catenin inhibitor	PFK115-584	AML [141] T-ALL [81]	Preclinical
β-catenin/TCF inhibitor	iCRT14	B-/T-ALL [142]	Preclinical
β-catenin inhibitor	BHX	CML [143]	Preclinical
β-catenin inhibitor	BC2059	AML [144]	Preclinical
